# Phase I/II Trial of Autologous Bone Marrow Stem Cell Transplantation with a Three-Dimensional Woven-Fabric Scaffold for Periodontitis

**DOI:** 10.1155/2016/6205910

**Published:** 2016-11-20

**Authors:** Shunsuke Baba, Yoichi Yamada, Akira Komuro, Yoritaka Yotsui, Makoto Umeda, Kimishige Shimuzutani, Sayaka Nakamura

**Affiliations:** ^1^Department of Oral Implantology, Osaka Dental University, 1-5-17 Otemae Chuo-ku, Osaka 540-0008, Japan; ^2^Department of Oral and Maxillofacial Surgery, Aichi Medical University School of Medicine, 1-1 Yazakokarimata, Nagakute, Aichi 480-1195, Japan; ^3^Department of Oral Radiology, Osaka Dental University, 1-5-17 Otemae Chuo-ku, Osaka 540-0008, Japan; ^4^Department of Periodontology, Osaka Dental University, 8-1 Kuzuhahanazono-cho Hirakata, Osaka 573-1121, Japan; ^5^Department of Biochemistry, School of Dentistry, Aichi Gakuin University, 1-100 Kusumoto-cyo, Chikusa-ku, Nagoya 464-8650, Japan

## Abstract

Regenerative medicine is emerging as a promising option, but the potential of autologous stem cells has not been investigated well in clinical settings of periodontal treatment. In this clinical study, we evaluated the safety and efficacy of a new regenerative therapy based on the surgical implantation of autologous mesenchymal stem cells (MSCs) with a biodegradable three-dimensional (3D) woven-fabric composite scaffold and platelet-rich plasma (PRP). Ten patients with periodontitis, who required a surgical procedure for intrabony defects, were enrolled in phase I/II trial. Once MSCs were implanted in each periodontal intrabony defect, the patients were monitored during 36 months for a medical exam including laboratory tests of blood and urine samples, changes in clinical attachment level, pocket depth, and linear bone growth (LBG). All three parameters improved significantly during the entire follow-up period (*p* < 0.0001), leading to an average LBG of 4.7 mm after 36 months. Clinical mobility measured by Periotest showed a decreasing trend after the surgery. No clinical safety problems attributable to the investigational MSCs were identified. This clinical trial suggests that the stem cell therapy using MSCs-PRP/3D woven-fabric composite scaffold may constitute a novel safe and effective regenerative treatment option for periodontitis.

## 1. Introduction 

Periodontitis is a highly prevalent disease well known to reduce the quality of life of middle-aged and older people [1]. This common chronic inflammatory disease is caused by the formation of a bacterial biofilm on the tissues supporting the mouth and teeth, leading to the progressive destruction of the tissues and the loss of the affected teeth. The formation of intrabony defects is a frequent complication of periodontitis, which is generally treated by functional periodontal regeneration involving restoration of the alveolar bone and new cementum [2]. Conventional nonsurgical periodontal treatment and/or open flap debridement can reduce pocket depth and inflammation. However, the functional regeneration of the lost periodontal tissue and the normal structure are insufficient [3].

Historically, *β*-tricalcium phosphate (*β*-TCP) and ceramics were used for regenerative approach, but these grafting procedures resulted in the formation of long junctional epithelium [4]. Systematic reviews revealed that bone replacement grafts (including autogenous bones and demineralized freeze-dried bone allografts) and guided tissue regeneration (GTR) [5] are effective methods for periodontal regeneration [4]. On the other hand, ongoing trials are exploring new treatments to accelerate the regeneration of periodontal tissue. The consensus is that biologics, such as enamel matrix derivatives (EMD) and recombinant human platelet-derived growth factor-BB (rhPDGF-BB) with *β*-TCP, enhance the effectiveness of periodontal regenerative therapies [2, 4]. Although these applications generally improve bone filling and the clinical parameters, complete regeneration is not achieved, particularly in cases with advanced periodontal defects [3]. Moreover, the cellular cementum regenerated by GTR is apparently different from the natural cementum formed during tooth development, whereas the cementum regenerated by EMD is acellular [6]. There are also concerns about the fact that EMD preparations are poorly characterized [4]. Accordingly, further theoretical and technical developments are required in the field of periodontal regenerative therapies.

Regenerative medicine is more attractive optional treatment for the regeneration of periodontal tissues and function. The main concept involves three elements: stem cells, scaffolds, and growth factors. The mesenchymal stem cells (MSCs) found within the periodontal tissues are multipotent cells that can replicate as undifferentiated cells and possess the capacity of multilineage differentiation [7–9]. They are more recent and highly regarded because of their potential for use in cell-based therapy for systemic diseases. However, very few studies reported clinical applications of regenerative medicine using stem cells for periodontal regeneration [10].

Our team performed several clinical studies using isolated autogenous MSCs with platelet-rich plasma (PRP) as a source of signal molecules and scaffolds for bone regeneration and succeeded in achieving bone formation in the grafted area [9, 11, 12]. PRP contains a variety of growth factors and is autologously modified fibrin glue. It is reported to promote early consolidation and graft mineralization. The growth factors include fibroblast growth factor (FGF), platelet-derived growth factor (PDGF), vascular endothelial growth factor (VEGF), and transforming growth factor-*β* (TGF-*β*), and they also stimulate wound healing through their effects on the proliferation and differentiation of various cell types [13].

A preclinical animal study indicated that MSCs also play an important role in the cementification process and the structure of the regenerated cementum was more similar to natural cementum of roots than to the one produced by GTR. A study using cell tracking assays with green fluorescent protein (GFP) demonstrated that implanted MSCs would survive and participate in cementum regeneration [9]. Thus, we conducted a small-scale pilot study using bone marrow-derived MSCs (BMMSCs) for the treatment of periodontal disease [11]. Although the results support the potential of BMMSCs for successful periodontal regeneration, more effective scaffolds are needed for cases of advanced periodontal disease.

Our team developed a three-dimensional (3D) woven-fabric scaffold composed of biodegradable poly-L-lactic acid resin fibers [14]. The porous basket-shaped material was designed to enhance bone regeneration by securing spaces in which implanted cells proliferate and differentiate as well as internal spaces retaining the gel-like graft material. Our previous preclinical study using the canine mandibles model demonstrated that this scaffold considerably accelerates regeneration of the bone and periodontal tissue in defects created on the tooth root aspect [14]. Therefore, here we present the results of a long-term clinical trial conducted to evaluate the safety, efficacy, and stability of a novel periodontal regeneration therapy using MSCs, PRP, and our woven-fabric scaffold.

## 2. Materials and Methods

### 2.1. Study Participants

Adult patients who visited the Institute of Biomedical Research and Innovation hospital (Kobe, Japan) for the treatment of chronic periodontitis were enrolled in this phase I/II clinical trial. The inclusion criteria were as follows: (1) age 35–60 years, (2) a probing pocket depth (PPD) ≥ 4 mm, (3) at least 10 teeth in the mandibular, (4) teeth for which recovery by an existing periodontal surgery is not to be expected, and (5) good general health without any sign of systemic disease. The exclusion criteria were as follows: smoking within the last 6 months before study enrollment and pregnancy. All patients included in this study received standard nonsurgical periodontal therapy. Afterward, teeth exhibiting significant vertical bone resorption on dental X-ray and periodontal pockets ≥ 4 mm were selected. Two healthy teeth per patient were used as the control. Verbal and written informed consents were obtained from the patients. This study conformed to STROBE Guidelines and was approved by the institutional review board of the Foundation for Biomedical Research and Innovation registered in ClinicalTrails.gov (NCT00221130, Supplemental Data available online at http://dx.doi.org/10.1155/2016/6205910).

### 2.2. Preparation of Bone Marrow-Derived Mesenchymal Stem Cells

BMMSCs were prepared as previously described [9, 11, 12] and the cells were prepared in cell processing center of Translational Research Informatics Center in Foundation for Biomedical Research and Innovation that was compliant with Good Manufacturing Practice guidelines. In brief, 1 month before the periodontal surgery, autogenous MSCs were harvested by aspirating the iliac bone marrow from each subject. Base media and low-glucose Dulbecco's modified Eagle medium containing growth supplements (50 mL serum, 10 mL 200 mM L-glutamine, and 0.5 mL penicillin-streptomycin mixture containing 25 IU penicillin and 25 *μ*g streptomycin) were purchased from Cambrex (Walkersville, MD). The supplements used to induce osteogenesis (dexamethasone, sodium *β*-glycerophosphate, and L-ascorbic acid 2-phosphate) were bought from Sigma Chemical (St Louis, MO). The presence of osteoblast-like cells was confirmed by measuring alkaline phosphatase (ALP) activity.

### 2.3. Preparation of Platelet-Rich Plasma (PRP)

Total hemocyte count was determined during the preoperative hematologic assessment, whereas a blood sample was obtained 1 day before the surgery. The blood was collected under sterile conditions in a bag containing anticoagulant citrate. The blood was centrifuged to separate the yellow plasma containing the platelets and the buffy coat containing the leukocytes. The plasma was centrifuged to consolidate the platelets into a pellet. The platelet-poor plasma and the plasma supernatant were discarded. The pellet of platelets and buffy coat/plasma fraction were resuspended into the remaining plasma used to prepare a platelet gel.

### 2.4. Cell Transplantation

**Figure 1:**
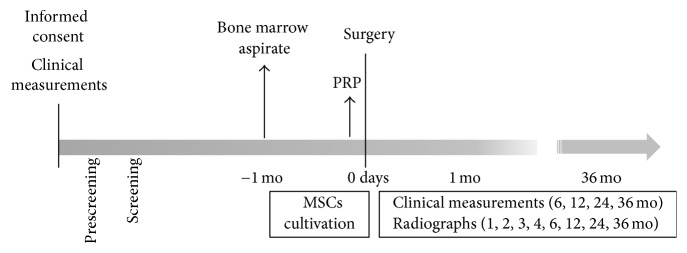
Treatment schedule.

The clinical trial was conducted according to the schedule shown in Figure 1. Before the surgery, each subject rinsed their mouth with 0.2% chlorhexidine solution. Then, the surgery was initiated under local anesthesia with xylocaine adrenaline 2% (Astra). To avoid interactions from the xylocaine, the injection was given at about a tooth's width. After making the pocket and releasing incisions, the full-thickness flap was elevated on both the buccal and lingual aspects, and the inner epithelium of the flap was removed. The granulation tissue was thoroughly removed in the area of the bone defect, and the tooth root was planned to smoothen the surface. No bone recontouring was performed. The mixture of BMMSCs and PRP, combined with human thrombin (5,000 units) dissolved in 10% calcium chloride, was perfused in a 3D woven-fabric composed of poly-L-lactic acid resin fibers [14], which was then placed around the tooth root. The flap was repositioned and the wound was closed by a suture. The suture was removed after 2 weeks. The subjects were instructed to rinse their mouth using solution of chlorhexidine digluconate three times a day. No mechanical cleaning was allowed on the surgical site during the first 4 weeks after surgery. After this period, professional mechanical tooth cleaning was performed once every 2 months.

### 2.5. Clinical Evaluation

The primary endpoints of this study were the clinical attachment level (CAL) and pocket depth (PD). CAL or PD were measured at each follow-up visit (postoperative 6-, 12-, 24-, and 36-month time-points), and the changes were calculated relative to the pretreatment measurements. The median of five measurements on each tooth was used for the efficacy analysis. Clinical mobility was based on the Periotest value, which was measured three times at the center of the buccal aspect of the tooth, and the mean was used in the analysis. Postoperative changes in vertical bone defects were assessed by X-rays at each follow-up visit (postoperative 1-, 2-, 3-, 4-, 6-, 12-, 24-, and 36-month time-points)and defined as the linear bone growth (LBG). The postoperative depth of the intrabony defect was calculated from the distance between the apex and cementoenamel junction on the preoperative and postoperative radiographs at the same magnification.

### 2.6. Safety Evaluation

Each follow-up visit included a medical exam of the entire body and oral cavity (postoperative 1- and 2-week and 6-, 12-, 24-, and 36-month time-points) as well as laboratory tests of blood and urine samples (postoperative 6-, 24-, and 36-month time-points). Adverse events were documented and analyzed for any temporal relationship with the surgical procedure and/or scaffold.

### 2.7. Statistical Analysis

The changes in CAL, PD, the depth of the intrabony defect, and clinical mobility before and after implantation were analyzed using a linear mixed model assuming a compound symmetry covariance structure. Multiple comparisons by the Dunnett-Hsu method were performed at each follow-up time-point using the preoperative value as the control.

## 3. Results

### 3.1. Transplantation of the BMMSCs-PRP/3D Woven-Fabric Composite Scaffold Gel

A total of 10 patients (three males and seven females; mean age: 48.4 years) completed the standard periodontal therapy and were enrolled in the study. The intrabony defects included one-wall defects at three sites, two-wall defects at six sites, and three-wall defects at one site.

**Figure 2:**
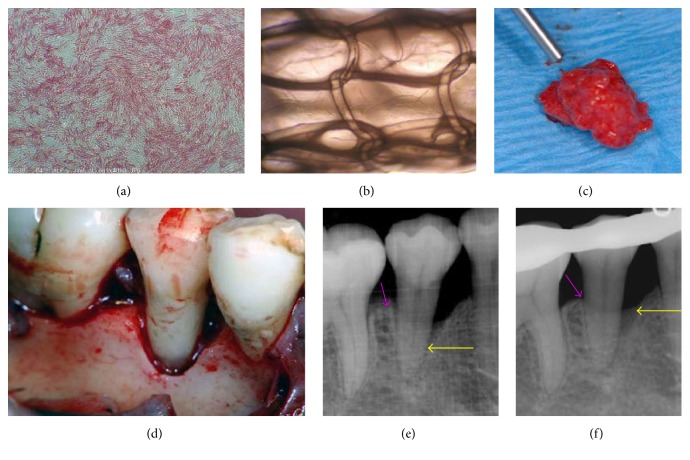
(a) Positive alkaline phosphatase staining of osteoinduced BMMSCs. (b) BMMSCs proliferate on the basket-shaped scaffold. (c) The combination of BMMSCs/PRP and thrombin in calcium chloride generates a gel-like structure. (d) Representative image of the periodontal intrabony defect (preoperative). (e, f) X-ray images taken before and 8 weeks after the surgery.

The characteristics of BMMSCs were assessed based on ALP staining and enzyme activity. After the induction of osteogenesis, the cells were positive for ALP staining (Figure 2(a)) and exhibited high ALP activity (BMMSCs: 2.01 units/well and induced osteoblast: 18.05 units/well), thereby establishing the osteogenic capacity of BMMSCs. The* in vitro* experiment demonstrated that BMMSCs could proliferate on the basket-shaped 3D woven-fabric composite scaffold used in this clinical study (Figure 2(b)).

When BMMSCs-PRP solution was combined with thrombin dissolved in a calcium chloride solution, the mixture adopted a gel-like texture (Figure 2(c)) in the 3D woven-fabric composite scaffold fitted in the periodontal bony defects. The mean cell number transplanted to each patient was 3.5 × 10^7^ cells. In PRP, the mean platelet count (1,352,600/*μ*L) was 490% higher than that at the baseline, which confirmed the sequestration ability of the scaffold.

### 3.2. Periodontal Inspections

The mean changes in CAL, PD, and clinical mobility are shown in Table 1. CAL decreased over time, and therapy efficacy was significant at each follow-up visit, based on the linear mixed model (each time-point: *p* < 0.0001). The multiple comparisons by the Dunnett-Hsu method revealed significantly different CAL at each follow-up time-point (*p* < 0.0001 at the 6-, 12-, 24-, and 36-month time-points), compared with the preoperative level. On the other hand, CAL of the control group increased significantly from the preoperative time-point to the postoperative 12- (*p* = 0.0076), 24- (*p* = 0.0019), and 36-month (*p* = 0.0001) time-points. In addition, PD of the test group was significantly lower during the entire follow-up period compared with the preoperative level (each time-point: *p* < 0.0001). In contrast, PD of the control group showed an increasing trend at each follow-up period.

The clinical mobility of the test group showed a decreasing trend after the surgery, but there was no significant efficacy for the control or test groups at any of the follow-up time-points, based on multiple comparisons by the Dunnett-Hsu method or Wilcoxon's signed-rank test. The control group showed no significant difference in clinical mobility at all time-points.

**Figure 3:**
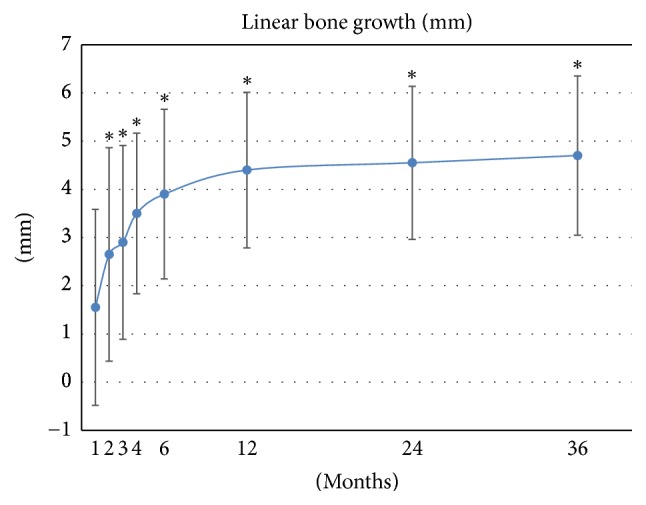
Mean change in the depth of the intrabony defect after operation. Linear bone growth (LBG) significantly increased during the follow-up period. ^*∗*^ *p* < 0.01.

The test group showed a significant decrease in the depth of the intrabony defect during the follow-up period (*p* < 0.0001) (Figures 2(d)–2(f) and 3). The multiple comparisons revealed significant improvement of the intrabony defect at the 2- (*p* = 0.0078), 3- (*p* = 0.002), 4- (*p* = 0.002), 6- (*p* = 0.002), 12- (*p* = 0.002), 24- (*p* = 0.0039), and 36-month (*p* = 0.0039) time-points, compared with the preoperative level.

### 3.3. Safety Evaluation

During the follow-up period, the overall plaque control record of the subjects remained <30%, indicating favorable oral hygiene practice (data not shown). The frequency of each adverse event observed throughout the follow-up period is shown in Table 2. The most frequent adverse event shown to have a causative relationship with the therapy was facial swelling (five cases), followed by gingival swelling (four cases) and angular cheilosis (three cases). All patients recovered spontaneously and completely from all adverse events. No patients have pain nor discomfort that had causative relationship with the therapy in intraoral region. In addition, no tooth loss was found after surgery during follow-up period. None of the abnormal laboratory test values obtained from blood and urine analyses were related to the therapy (data not shown).

## 4. Discussion

Conventional treatments of periodontitis may reduce the progression of periodontitis, but they generally fail to restore the normal periodontal supporting structures that were damaged by the disease [15]. Tissue engineering involves three strategies adopted for the creation of new tissue, isolated stem cells, signal molecules, and biocompatible scaffolds [16]. Regenerative medicine by autogenous cell transplantation is considered among the most promising therapeutic concepts currently developed because it solves several issues, namely, the morbidity at the donor site due to autologous grafts, immunogenicity of the allogenic grafts, and loosening of the alloplastic implants. Scaffolds and growth factors have been applied to the engineering concept of periodontal treatments, but few studies tested the potential of their combined effects in clinical trials [11]. Furthermore, successful periodontal regeneration requires the addition of progenitor cells that can proliferate and differentiate into specialized cells of regenerative capacity [17]. Many animal studies provided evidence that MSCs can be safe and effectively used to support periodontal regeneration [3]. The present study proposes a treatment combining all three elements: BMMSCs, a 3D poly-lactic-acid-based synthetic material, and a PRP solution of autologously modified fibrin glue and growth factors.

A study assessing the outcome of conventional periodontal surgery for periodontitis reported gains in CAL of 0.2–1.5 mm, reductions in PD of 1.5–2.7 mm, and LBG of 0.3–1.1 mm, measured 12 months after the procedure [18]. In contrast, the unique composition of the mixed transplants that we developed led to >twofold higher gains in CAL (2.68 mm) as well as higher reductions in PD (2.48 mm) and fourfold higher LBG (4.4 mm) 12 months after the procedure. Furthermore, our study was designed to monitor these three parameters over a period of 36 months to determine the linearity and persistence of these improvements. The analysis showed a gradual improvement in all three parameters over at least 36 months.

Previous clinical studies incorporated growth factors into their scaffold to stimulate periodontal regeneration, but they consistently yielded lower LBG values compared to those observed in the present study: rhPDGF-BB/*β*-TCP (3.3 mm at 24 months) [19], rh-FGF-2/*β*-TCP (3.7 mm at 6 months) [20], rh-FGF2/hydroxypropyl cellulose (1.9 mm at 9 months), or EMD (1.3 mm at 9 months) [21]. Altogether, these data demonstrate the importance of stem cell therapy for an effective periodontal regeneration. This finding is supported by animal studies that showed BMMSCs differentiating into periodontal cells to regenerate periodontal tissues [9, 22]. Furthermore, the signaling molecules contained in PRP may stimulate cell migration, proliferation, and differentiation.

Our clinical trial included an interview, whole-body physical examinations, extraoral and intraoral reaction tests, and clinical monitoring for potential adverse events related to this regenerative medicine therapy. There was no relationship between the stem cell therapy and frequency of the adverse events, which were all normal reactions to periodontal surgery. In addition, all patients spontaneously and completely recovered from all adverse events, without any specific treatment. These results are comparable to the previous studies that indicated no adverse reactions after local cell administration [9–12, 22]. Moreover, none of the abnormal laboratory test results, obtained from blood or urine samples, were related to the therapy. The scaffold materials were autogenous in origin basically and/or biodegradable. Previously, we performed the safety assessment of the cultured BMMSCs and no mycoplasma infection was detected and no tumorigenesis was found. In addition, flow cytometry analysis showed that the BMMSCs were positive for MSC markers and negative for hematopoietic lineage and monocytic markers [9]. These data would indicate that cultured BMMSCs used in this study were safe and possessed characteristics of MSCs. Collectively, these data would support the safety of this new effective treatment of periodontitis.

Since the main limitation of this study is the small sample size, multicenter, well-designed randomized controlled trials are needed to confirm the efficacy and safety of this treatment. Further investigation is required to develop more suitable scaffolding materials in an attempt to treat more severe periodontal defects. Also, the outcome of periodontal regeneration is negatively affected by poor oral hygiene [2]. In the present study, the overall plaque control record of the subjects remained <30% throughout the follow-up, indicating favorable oral hygiene practice. This factor probably contributed to the successful periodontal regeneration and should be closely monitored during future large-scale studies assessing the potential of this new therapy.

## 5. Conclusion

Long-term clinical trials, including a follow-up period of at least 3 years, are preferable to monitor the regeneration of periodontal tissues based on all key factors, namely, CAL, PD, and LBG. However, very few long-term clinical trials have been reported. The present 3-year trial showed that our new BMMSCs-PRP/3D woven-fabric composite scaffold gel was safe and provides significant long-term improvements in all clinical endpoints and efficient regeneration of the true periodontal tissue. Therefore, this new stem cell periodontal regenerative therapy may constitute a new promising regenerative treatment option for periodontal diseases. Future more rigorous clinical trials are recommended to determine safety and efficacy of the stem cell-based periodontal therapy.

## Supplementary Material

The study protocol.

## Figures and Tables

**Table 1:** Clinical outcome.

	Mean change (SD)				*p* value
	6 months	12 months	24 months	36 months	
CAL regained (mm)					
Test	2.72 (0.92)	2.68 (1.017)	3.09 (1.073)	3.24 (0.857)	<0.0001
Control	−0.52 (0.766)	−0.95 (0.852)	−0.84 (0.94)	−1.06 (1.125)	0.0004
Pocket depth changed (mm)					
Test	−2.46 (1.052)	−2.48 (0.998)	−3.02 (0.854)	−3.16 (0.583)	<0.0001
Control	0.33 (0.789)	0.73 (0.880)	0.52 (1.041)	0.68 (1.127)	0.0512
Mobility (Periotest value)					
Test	−1.36 (4.856)	−1.5 (3.095)	−1.87 (9.351)	−4.62 (7.567)	0.4802
Control	−0.34 (4.461)	−0.44 (5.623)	2.34 (9.152)	−0.01 (6.193)	0.8104

**Table 2:** Adverse event.

Extraoral reactions	Number of events	Outcome		
		remain	resission	healing
Pain				
Lumbar	1	0	0	1
Tenderness				
Cheek	1	0	0	1
Swelling				
Face	5	0	0	5
Submandibular lymph nodes	1	0	0	1
Lumbar	1	0	0	1
Abnormal perception	1	0	0	1

Intraoral reactions				

Pain	0	0	0	0
Bleeding	1	0	0	1
Gingival swelling	4	0	1	3
Aesthetic problem of gingiva	1	0	0	1
Redness of gingiva	2	0	0	2
Hematoma	1	0	0	1
Hyperaesthesia	2	0	0	2
Angular cheilosis	3	0	0	3
Canker sore	0	0	0	0
Gingival abscess	0	0	0	0
Tooth mobility	0	0	0	0
Discomfort	0	0	0	0
