# Supplementary material for: Phase I/II Trial of Autologous Bone Marrow Stem Cell Transplantation with a Three-Dimensional Woven-Fabric Scaffold for Periodontitis
Source: Stem Cells Int. 2016 Nov 20;2016:6205910. doi: 10.1155/2016/6205910 (PMC5136404; doi:10.1155/2016/6205910)
Supplement: Supplementary file 1 — The study protocol. [file 6205910.f1.doc]

**Study Protocol**

| **Descriptive Information** | |
| --- | --- |
| **Brief Title  ^ICMJE^** | Clinical Trials of Regeneration for Periodontal Tissue |
| **Official Title  ^ICMJE^** | Clinical Trial of Regenerative Periodontal Tissue by Transplanting Mesenchymal Stem Cells and Osteoblast Cells - I, II Phase- |
| **Brief Summary** | Adult periodontitis is a chronic infective disease affecting the periodontium. Periodontitis induce the destruction of attachment apparatus of teeth, resulting in periodontal pocket formation and teeth loss. This study will test the safety and efficacy of alveolous bone reproduction by the transplantation of mixture named periodontium injectable gel for the adult periodontitis patients. Injectable gel is the mixture of ex-vivo cultured mesenchymal stem stem cells, ex-vivo cultured osteoblast-like cells differentiated from mesenchymal stem cells and scaffold (include, platelet rich plasma, human thrombin and calcium chloride). |
| **Detailed Description** | Periodontal disease is an infectious disease. Gingivitis and periodontitis are the 2 major forms of this. Their primary etiology is bacterial plaque, which can induce destruction of the periodontal apparatus. Gingivitis is inflammation of the gingival that does not result in clinical attachment loss. Periodontitis is inflammation of the gingival and is characterized by loss of connective tissue attachment and alveolar bone.  Therapeutic approaches for periodontitis are divided into two categories: 1) anti-infective treatment; 2) regenerative therapy.  Several surgical techniques have been developed to regenerate periodontal tissues including guided tissue regeneration, bone grafting, the use of enamel matrix derivative. However, these techniques are not reached for complete regeneration of the periodontium.  This study will test the safety and efficacy of alveolous bone reproduction by the transplantation of mixture named periodontium injectable gel for the adult periodontitis patients. Injectable gel is the mixture of ex-vivo cultured mesenchymal stem stem cells, ex-vivo cultured osteoblast-like cells differentiated from mesenchymal stem cells and scaffold (include, platelet rich plasma, human thrombin and calcium chloride). |
| **Study Type  ^ICMJE^** | Interventional |
| **Study Phase** | Phase 1 Phase 2 |
| **Study Design  ^ICMJE^** | Allocation: Non-Randomized Endpoint Classification: Safety/Efficacy Study Intervention Model: Single Group Assignment Masking: Open Label Primary Purpose: Treatment |
| **Condition  ^ICMJE^** | Adult Periodontitis |
| **Intervention  ^ICMJE^** | Procedure: surgical operation of cell transplantation |
| **Recruitment Information** | |
| **Enrollment  ^ICMJE^** | 10 |
| **Eligibility Criteria  ^ICMJE^** | Inclusion Criteria:  1.Patient with adult periodontitis. 2. There are ４mm or more pocket using . 3.There are ten existing tooth or more of the lower jaw. 4.The brushing instruction is received, and the plaque control is maintained excellently. 5.The recovery by an existing periodontal operation cannot be expected. 6.The age is from 35 to 60 years old. 7.Blood clot function is normal. 8.The liver function is normal. 9.The intention and the ability of going to hospital regularly are possessed. 10.Agreement by the document is obtained.  Exclusion Criteria:  1.Patient who has acute symptom of periodontitis. 2.Patient that decayed tooth is on teeth or the next teeth to be treated. 3.Patient who has contracted diabetic or autoimmune disease. 4.Patient who has contracted infectious disease. 5.Patient who has contracted osteoporosis. 6.Patient who has smoking habit within six months before it registers. 7.Patient who is taking treatment of hypertension and/or epilepsy. 8.Patient who has pregnancy or doubt of pregnancy. 9.Patient who has heavy allergic disease. |
| **Gender** | Both |
| **Ages** | 35 Years to 60 Years (Adult) |
| **Accepts Healthy Volunteers** | Yes |
| **Listed Location Countries  ^ICMJE^** | Japan |
| **Administrative Information** | |
| **NCT Number  ^ICMJE^** | NCT00221130 |
| **Other Study ID Numbers  ^ICMJE^** | BRI PDT03-01 |
| **Study Sponsor  ^ICMJE^** | Translational Research Informatics Center, Kobe, Hyogo, Japan |
